# Perioperative management of thyroglossal duct cystectomy in a pediatric patient: A case report

**DOI:** 10.1002/ccr3.3607

**Published:** 2020-12-04

**Authors:** Aiji Sato (Boku), Eisuke Kako, Nozomi Okuni, Nobuyoshi Kusama, Yuji Kamimura, Yoshiki Sento, MinHye So, Motoshi Tanaka, Hironori Miyamoto, Shinichiro Kato, Masaki Kobayashi, Yasuyuki Shibuya, Kazuya Sobue

**Affiliations:** ^1^ Department of Anesthesiology Aichi Gakuin University School of Dentistry Nagoya Japan; ^2^ Department of Anesthesiology and Intensive Care Medicine Nagoya City University Graduate School of Medical Sciences Nagoya Japan; ^3^ Department of Oral and Maxillofacial Surgery Nagoya City University School of Medical Sciences Nagoya Japan

**Keywords:** airway obstruction, dexmedetomidine, difficult airway management, pediatric patient, thyroglossal duct cysts

## Abstract

Thyroglossal duct on the dorsum of the tongue in the pediatric patient can cause a difficult airway due to the large mass and risk of airway obstruction associated with a swollen tongue after surgery.

## Graphical abstract

Careful airway management in the perioperative period contributed to the prevention of the occurrence of respiratory complications.

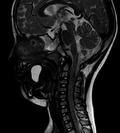

## 1 INTRODUCTION

Thyroglossal duct on the dorsum of the tongue in pediatric patients can cause a difficult airway due to the large mass and risk of airway obstruction associated with a swollen tongue postoperatively.

Thyroglossal duct cysts arise from remnants of the embryonic thyroglossal duct and commonly develop in the median cervix. Its basic treatment is to resect the cyst along with the fistulous tract.^1^ Herein, we report the perioperative management of a pediatric patient with thyroglossal duct on the dorsum of the tongue for thyroglossal duct cystectomy predicted to cause dyspnea in the perioperative stage.

Written consent was obtained from the patient's parents for this report.

## 2 CASE REPORT

**FIGURE 1:**
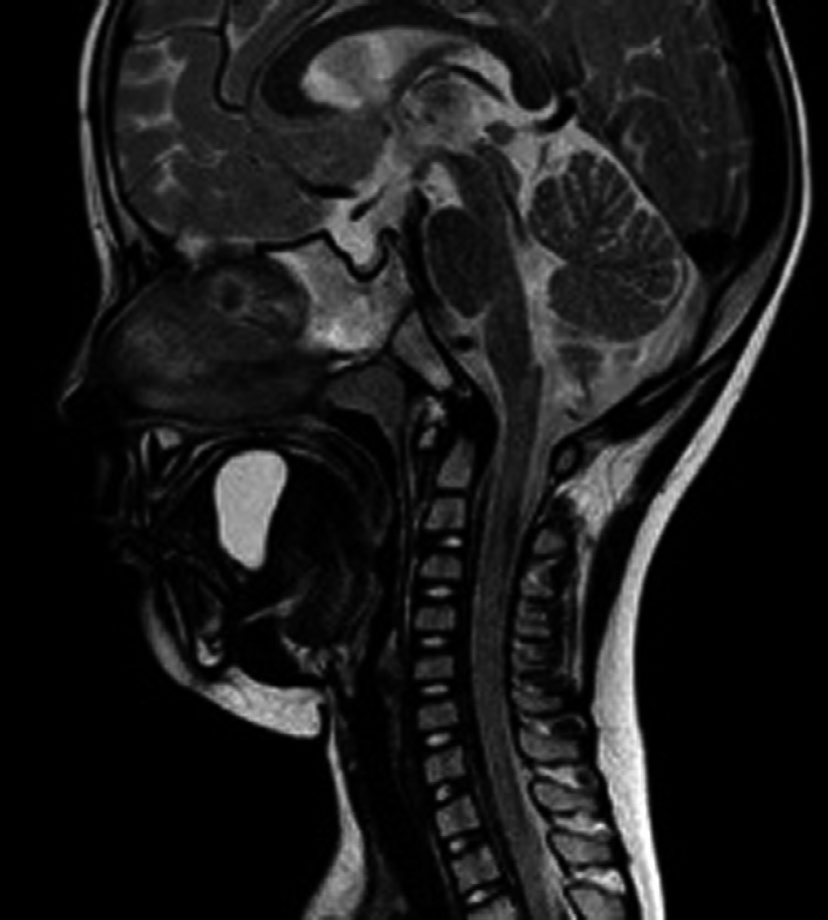
Magnetic resonance images. Sagittal section/a cystic lesion with clear margins is observed

**FIGURE 2:**
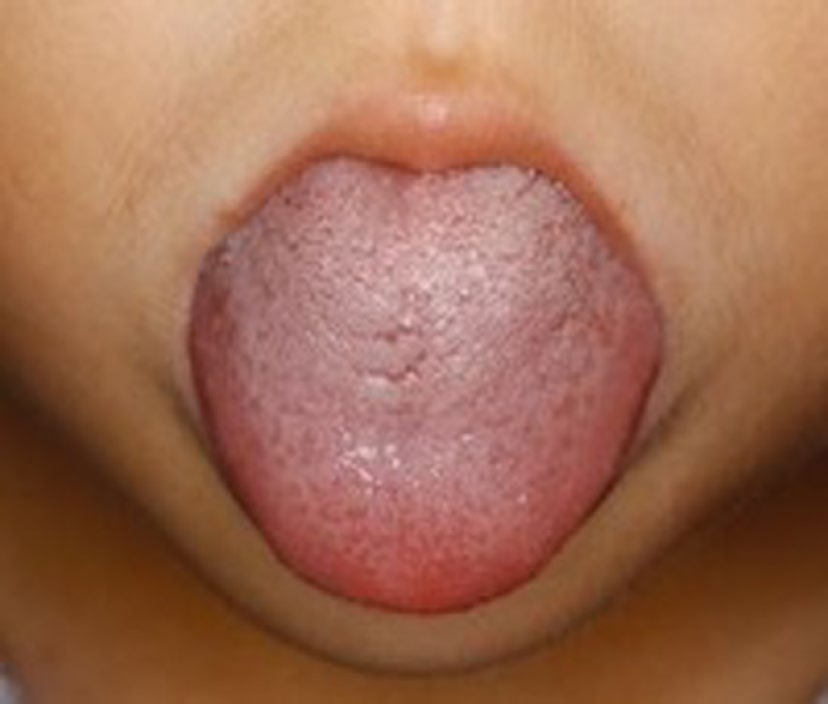
A large mass at the dorsum of the tongue is detected

A 5‐year‐old boy with a height of 110 cm and body weight of 18 kg complained of a mass detected at the middle dorsum of the tongue, and thus, he was referred to the Department of Oral Surgery of Nagoya City University Hospital by a family dentist. The patient would snore when laid on his back while sleeping. Detailed examination revealed a 35 × 25 × 25‐mm mass lesion on the dorsum of the tongue (Figures 1 and 2). His medical history showed that although he was being followed up by a local pediatrician for congenital adrenal hyperplasia, the follow‐up had been completed at the time of this operation. The patient had no allergies or any family history of diseases, and preoperative examination revealed no particular findings. Under general anesthesia, resection of the mass lesion located at the dorsum of the tongue was planned.

Regarding anesthesia management, these two concerns were considered: (a) a difficult airway due to the large mass present on the tongue and (b) the risk of airway obstruction associated with a swollen tongue postoperatively.

Before anesthetizing the patient, a peripheral intravenous route was secured on the left forearm to prepare for the difficult airway that was expected after the induction of anesthesia. Prior to the induction of anesthesia, oxygenation was administered with 100% oxygen for approximately 3 minutes. Anesthesia was induced with 6 L/min of oxygen and 6% sevoflurane. After the onset of anesthesia, mask ventilation temporarily became difficult due to the tongue mass and glossoptosis; however, mask ventilation was feasible after administering 15 mg of rocuronium and using an oral airway. Decreased peripheral capillary oxygen saturation associated with temporary dyspnea was not observed. Subsequently, 30 μg of fentanyl was administered, and after the muscle relaxant showed its effect, nasotracheal intubation was performed using the McGRATH™ MAC (McGRATH). The dorsal mass of the tongue was mobile. Hence, the tongue could not be completely removed from the field of view due to the mobile mass although the tongue has been removed from the field of view during intubation. However, the view with McGRATH showed a Cormack–Lehane Grade 2, and intubation was successfully performed during the first attempt. Anesthesia was maintained at 1 L/min of oxygen, 2 L/min of air, 2% sevoflurane, and 0.1‐0.2 μg/kg/min of remifentanil. Intraoperatively, 2 mg of dexamethasone was administered to prevent postoperative tongue swelling, and 200 mg of acetaminophen was administered for postoperative analgesia. Intraoperative findings included no tongue swelling at the end of the surgery. Therefore, extubation was considered possible in the operating room. After completing the surgery, 50 mg of sugammadex, a muscle relaxant antagonist, was administered, and the patient was extubated after confirming body movement, eye opening, and sufficient spontaneous respiration. After extubation, no problems were observed with the patient's respiratory condition. The total surgical duration was 1 hour 27 minutes, with the time spent under anesthesia of 2 hour 13 minutes and amount of blood loss of 6 g.

In the postoperative intensive care unit (ICU), dexmedetomidine (0.4 μg/kg/hr) was administered for sedation, and additional dexamethasone was administered to prevent tongue swelling. The patient was managed in the ICU until the next morning, but no tongue swelling was observed. Then, the patient was discharged from the ICU due to stable respiratory status and vital signs.

## 3 DISCUSSION

**FIGURE 3:**
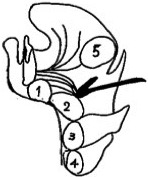
Vertical site of the onset of thyroglossal duct cyst, adapted from reference^4^ with modifications. 1. Mentum‐type, 2. Hyoid bone‐type, 3. Sublingual‐type, 4. Thyroid‐type, 5. Base of the tongue type

Cysts on the tongue are one of the major diseases that cause upper airway obstruction in pediatric patients.^2^ Their origin varies, including those derived from the thyroglossal or lingual ducts, cystic degeneration of the lingual thyroid, retention cysts arising from the mucous glands at the base of the tongue, and cysts generated from remnant cells during the embryonic stage.^3^ In this report, a thyroglossal duct cyst was detected based on the postoperative histopathological results, and the common sites are shown in Figure 3.^4^

First, this case has a difficult airway management. Kumanomido et al^2^ described that the need for tracheostomy and subglottic space, the presence/absence of a tracheal shift, mandibular size, and the need for fiberscope‐guided intubation should be assessed to evaluate the difficulty of tracheal intubation according to the cyst size. The possibility of tracheostomy was excluded in our patient because of the cyst location, that is, near the dorsum of the tongue and anterior to the typical site of origin, and with snoring as the only daily symptom reported. Furthermore, the patient aged only 5 years; therefore, conscious fiberscope‐guided intubation was not considered feasible. Moreover, preoperative computed tomography images revealed no subglottic space or tracheal deviation, and the patient did not present with micrognathia. Therefore, slow induction with sevoflurane was performed as usual, with emergency airway maintenance preparation. A peripheral intravenous route was secured before the induction of anesthesia to prepare for the expected difficult airway. After the onset of anesthesia, mask ventilation was difficult temporarily due to the effects of the tongue mass and glossoptosis; however, mask ventilation was sufficiently performed using muscle relaxants to facilitate an effective oral airway. Mask ventilation would have still been difficult if the cyst would have been localized near the base of the tongue.

For tracheal intubation, McGRATH was used in our patient. Its structure is similar to that of a conventional Macintosh laryngoscope; therefore, tracheal intubation is possible under direct vision using the display mounted on top of McGRATH handle only.^4^ McGRATH has been used for the orotracheal intubation in both normal patients and patients expected to have difficult intubation and has been reported to provide improved glottis visibility during tracheal intubation and an increased tracheal intubation success rate.^5^, ^6^ Its utility has been reported in pediatric patients.^7^, ^8^, ^9^ An airway scope using pediatric Intlock blades is useful for pediatric patients with difficult airways.^10^ However, McGRATH was used for intubation in this patient because of its thin blade and good operability. Although the tongue could not be completely excluded using McGRATH, its Cormack–Lehane view was Grade 2, and thereby, intubation was successfully performed on the first attempt without any issues.

The second problem in this case is airway obstruction associated with postoperative tongue swelling. In this case, intraoperative findings included no fistulous tract connected to the cyst, no resection of the hyoid bone, and no tongue swelling at the end of the surgery. Therefore, the patient was extubated in the operating room; however, as a precaution, the patient's respiratory condition was observed under sedation with dexmedetomidine in the ICU. In Japan, dexmedetomidine for the sedation of nonintubated pediatric patients is used since 2018. Without intubation, respiratory depression is a major issue, particularly in pediatric sedation. Dexmedetomidine does not have a less respiratory depressant effect and is also used for postoperative sedation in pediatric patients.^11^ In our patient, respiratory depression through sedation was suspected; however, the patient was kept on bed rest to avoid tongue swelling postoperatively. Therefore, dexmedetomidine was used. In addition, dexmedetomidine has analgesic effects, which to some degree seem to have contributed to the patient being on bed rest.

## 4 CONCLUSIONS

Thus, we report our experience in performing thyroglossal duct cystectomy on a pediatric patient predicted to experience perioperative airway maintenance and respiratory management difficulties due to a thyroglossal duct cyst. Although a difficult airway was expected due to the large tongue mass, no problems with airway maintenance occurred. Based on surgical findings, extubation in the operating room was feasible, and appropriate sedation using dexmedetomidine in the ICU prevented the occurrence of airway obstruction associated with tongue swelling.

## CONFLICT OF INTEREST

None declared.

## AUTHOR CONTRIBUTIONS

AS, NO, and EK: helped write this manuscript and the anesthetic management. NK, YK, YS, MS, and MT: helped with the anesthetic management and development of the overall anesthetic plan. MH, SK, and MK: helped with the operation and in writing this manuscript. YS: helped with the operation and supervised the writing of this manuscript. KS: helped with the supervision of the manuscript and development of the overall anesthetic plan.

## CONSENT FOR PUBLICATION

Written and signed consent was obtained from the patient's parents.
